# Natural Selection in Man

**Published:** 1911-12-09

**Authors:** 


### NATURAL SELECTION IN MAN.

The studies in eugenics carried on in Professor Pearson's laboratory, on previous occasions have been the subject of comment in The Hospital, and the latest publication from that laboratory touches on points as interesting to public health workers, doctors, and the community at large, as any of its predecessors. In it Mr. Snow subjects to mathematical analysis enormous masses of data dealing with birth and death, in order to determine if possible whether Darwinian natural selection has, in fact, been operating in England in the last generation or two. He criticises severely the supplement to the report of the Local Government Board, and arrives at conclusions diametrically opposite to those of Mr. Yule, whose opinions were adopted with less caution in the Report.

Briefly, the Local Government Board investigators suggest that natural selection is almost wholly inoperative in civilised society. That is, that an excessive infant mortality does not kill off, in the main, the weaklings; and this conclusion is reached by considering the mortality figures for ages one to five years, along with those of babies under one year, in the same districts. Mr. Snow very pertinently shows that in this comparison the statistician took the figures relating to ages one to five *for the same year* as those of the nurslings, and thus invaliditated his findings at the outset, since obviously he was not dealing with the survivors of a given year of infant birth and mortality. Natural selection, he affirms, in the form of a selective death-rate, is strongly operative in the early years of life. By this is meant that a high mortality in the first two years of life is followed by a low mortality in childhood, and conversely. In other words, a great reduction of infant mortality by improved hygienic administration results in the survival of a number of weaklings, who simply go to the wall during the next few years of life. It is pointed out that while marriage is just as prevalent among weak stocks as among the vigorous, and the former are as fertile as the latter, a proportion of the infants born each year are bound to be weaklings. True infantile mortality—as distinct from the large remediable death-rate from neglect, accident, ignorance of the mothers, etc.—finds most victims among this class.

Though this conclusion is given with due emphasis, and the opposite opinion of the Local Government Board advisers is said to be founded on wholly insufficient evidence, Mr. Snow does not of course decry efforts to reduce infant mortality; but he holds that the probable consequences of such efforts, as far as past experience can indicate them, should be completely understood. The "weeding out" process, as it at present works, is not uniform, because other factors than defective inherited constitutional power exert great influence. Thus in Glamorganshire infant mortality is more than twice as great as in Oxfordshire; not wholly because of any constitutional superiority of the inhabitants of the latter county, but because of the greater prevalence of neglect, insanitary sorroundings, and ignorance in the South Wales mining districts. The problem for the future is regarded by this author as not, *does* natural selection operate in man, but *how* does it operate? It is hoped that some answer to this may be rendered possible by aid of the results accruing from the medical inspection of school children.

